# Quantification of Health by Scaling Similarity Judgments

**DOI:** 10.1371/journal.pone.0089091

**Published:** 2014-02-21

**Authors:** Alexander M. M. Arons, Paul F. M. Krabbe

**Affiliations:** 1 Department for Health Evidence, Radboud University Medical Center, Nijmegen, The Netherlands; 2 University of Groningen, University Medical Center Groningen, Department of Epidemiology, Groningen, The Netherlands; Queensland University of Technology, Australia

## Abstract

**Objective:**

A new methodology is introduced to scale health states on an interval scale based on similarity responses. It could be well suited for valuation of health states on specific regions of the health continuum that are problematic when applying conventional valuation techniques. These regions are the top-end, bottom-end, and states around ‘dead’.

**Methods:**

Three samples of approximately 500 respondents were recruited via an online survey. Each sample received a different judgmental task in which similarity data were elicited for the top seven health states in the dementia quality of life instrument (DQI). These states were ‘111111’ (no problems on any domain) and six others with some problems (level 2) on one domain. The tasks presented two (dyads), three (triads), or four (quads) DQI health states. Similarity data were transformed into interval-level scales with metric and non-metric multidimensional scaling algorithms. The three response tasks were assessed for their feasibility and comprehension.

**Results:**

In total 532, 469, and 509 respondents participated in the dyads, triads, and quads tasks respectively. After the scaling procedure, in all three response tasks, the best health state ‘111111’ was positioned at one end of the health-state continuum and state ‘111211’ was positioned at the other. The correlation between the metric scales ranged from 0.73 to 0.95, while the non-metric scales ranged from 0.76 to 1.00, indicating strong to near perfect associations. There were no apparent differences in the reported difficulty of the response tasks, but the triads had the highest number of drop-outs.

**Discussion:**

Multidimensional scaling proved to be a feasible method to scale health-state similarity data. The dyads and especially the quads response tasks warrant further investigation, as these tasks provided the best indications of respondent comprehension.

## Introduction

Comprehensive and generic health-related quality of life (HRQoL) measures have been designed to capture an individual’s health status in a single value (index or weight). While mostly applied in cost-effectiveness analyses [1], [2], such values can also be used in health-outcomes research, disease-modeling studies, and monitoring of public health programs.

The most frequently used valuation techniques to derive health-state values are the standard-gamble (SG) [3], time trade-off (TTO) [4], and the visual analogue scale (VAS) [5], [6]. However, there are drawbacks to each of these traditional techniques, both theoretical and empirical. SG values tend to be biased by risk aversion and the SG task was often considered as too cognitively demanding [7]. TTO values incorporate time preferences in addition to health-state preferences [8]. In addition, difficulties arise when valuing states that are worse than ‘dead’, and some people are unwilling to trade any life years because they consider life worth living under any conditions [9], [10]. Even the new protocols (lead-time TTO, lag-time TTO, composite TTO) that were designed to overcome some of the problems of the traditional TTO protocol are subject to these biases [11]–[14]. Moreover, the TTO tasks have been framed in several ways, multiple iteration procedures have been applied, and the time horizons have differed. The VAS, which was introduced in the field of psychology, has been criticized for its interval properties [15], potential anchoring effects, and context and end-aversion biases [16]. The person trade-off method has been applied in the setting of public health evaluation, where the shortcomings of complex trade-off valuation techniques have been recognized, leading to the adoption of an easier ordinal response task [17].

Over the past decade, (discrete) choice models have gained considerable attention as an alternative to these conventional techniques [18]–[24]. Choice models are an extension to Thurstone’s law of comparative judgment (LCJ) [25]. Whereas Thurstone’s LCJ allows only the estimation of the relative values of health states based on paired comparisons [26], modern choice models extend it by regressing the relative weights of the domain levels that are part of the health-state descriptions [20], [27], [28]. These models are grounded in random utility theory, an idea that originated in psychology [25] and was subsequently adopted by economists [18]. A benefit is that the response tasks (e.g., paired comparisons) are cognitively less demanding, since they involve one of the most basic human operations, namely discrimination. Nonetheless, even though discrimination is one of the easiest response tasks for individuals, the operation is still limited by cognitive resources [29]. As such, even in paired comparisons the amount of information needs to be constrained.

**Figure 1:**
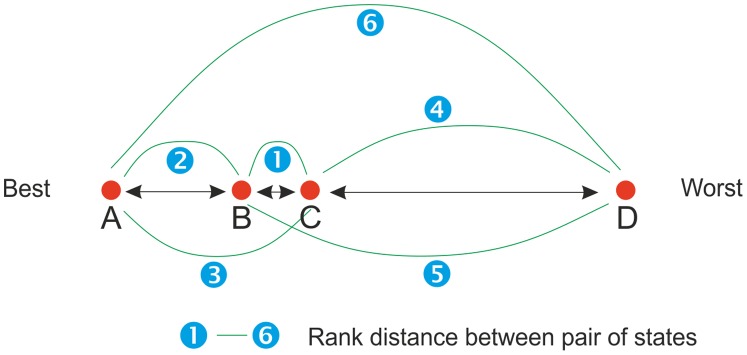
Schematic representation of the psychological distances and ranks between pairs of health states (input for non-metric MDS).

A serious drawback of the DC models is that relative distances between health states are produced. In many applications, however, absolute values are required. In particular if health-state values are used in computing conventional quality-adjusted life years (QALYs) an important requirement is that position of ‘dead’ (value = 0) is specified. Another related problem exists at the top end of the health-state continuum. For instance, ‘perfect health’ (or its synonym) is always preferable (dominant) in DC tasks. As a result, the health states closely positioned to ’full health’ cannot be accurately estimated. A methodology that has received little attention [30] and that may be able to deal with the limitations associated with DC modeling is (non-metric) multidimensional scaling (MDS) [31]–[33]. MDS is a collection of mathematical (hence, not statistical) techniques that can be used to analyze distances between objects (e.g. health states). These distances may be interval (metric) or rank distances (non-metric). For example, the ‘psychological distance’ between health states would be the perceived similarity between them, as elicited in specific judgmental tasks. MDS models similarity data as distances among pairs of health states in a geometric space. This is illustrated in Figure 1, which displays four health states with an interval distance between them represented by the length of the arrows. If we approximate the distances between the pairs of health states, we can use these rough estimates as input to infer the actual distances, which is done with metric multidimensional scaling. Conversely, when the distances are elicited as or converted into rank distances (the blue numbers in Figure 1), we can use non-metric multidimensional scaling. A benefit of non-metric MDS is that it allows responses that are less precise. As such, easier response tasks can be used to obtain this type of similarity data.

When a unidimensional solution (a basic requirement for measurement) is found with MDS, the health states are represented on an interval scale. The values can then be rescaled to a ‘0’ (dead) – ‘1’ (perfect health) scale. In theory, MDS is an elegant and robust method [34]–[38]. In practice, however, it might be very demanding at the data collection stage. For the time being, MDS seems more suited for exploring and deriving distances (quantification) in specific regions [30] or situations where conventional valuation techniques and choice models are not feasible or even fail. The current study is as an explorative study that attempts to investigate the feasibility of eliciting similarity data for health states and quantifying these states with MDS, meanwhile explaining in detail the procedures that underlie the approach.

## Methods

### Respondents

A company for marketing research (Survey Sampling International, Rotterdam) recruited the respondents for this study by selecting 1500 individuals aged 18–65 years from its respondent panel. After quota sampling, the sample was deemed roughly representative for the Dutch population with regard to age, gender, and education. An invitation was sent to the members of the sample by e-mail. Upon accepting, they were redirected to an online survey and then randomized to participate in one of three different response tasks (see below).

### Ethics Statement

The Dutch medical research involving human subjects act states the following regarding survey research: “No ethical approval is required unless: 1) subjects are under 18 years old, or are (mentally) incompetent 2) given the condition of the subject the survey is psychologically burdensome 3) subjects receive surveys on multiple occasions 4) subjects must travel or impose additional costs.” The current research project was sent to the local ethics committee (http://www.cmoregio-a-n.nl/) which concluded that it did not require ethical approval.

### Health States

The dementia quality of life instrument (DQI) [39] describes dementia-specific HRQoL in six domains: 1) physical health; 2) self-care; 3) memory; 4) social functioning; 5) mood; and 6) orientation. Each is measured on just three levels: 1) no problems; 2) some problems; or 3) severe problems. The DQI is intended for use among community-dwelling people. A particular health state is expressed as a six-digit number. The position of the digit denotes the domain, while the digit itself represents the level of problems in that domain. For example, ‘333332’ corresponds to a health state with severe problems in all domains except orientation, where there are some problems. In the present study, only the top end of the health-state continuum was investigated. Therefore, the following seven DQI health states were used: ‘111111’, ‘211111’, ‘121111’, ‘112111’, ‘111211’, ‘111121’, and ‘111112’. The DQI was chosen as this study was part of a research project for the development of the DQI.

### Response Tasks and Designs

**Figure 2:**
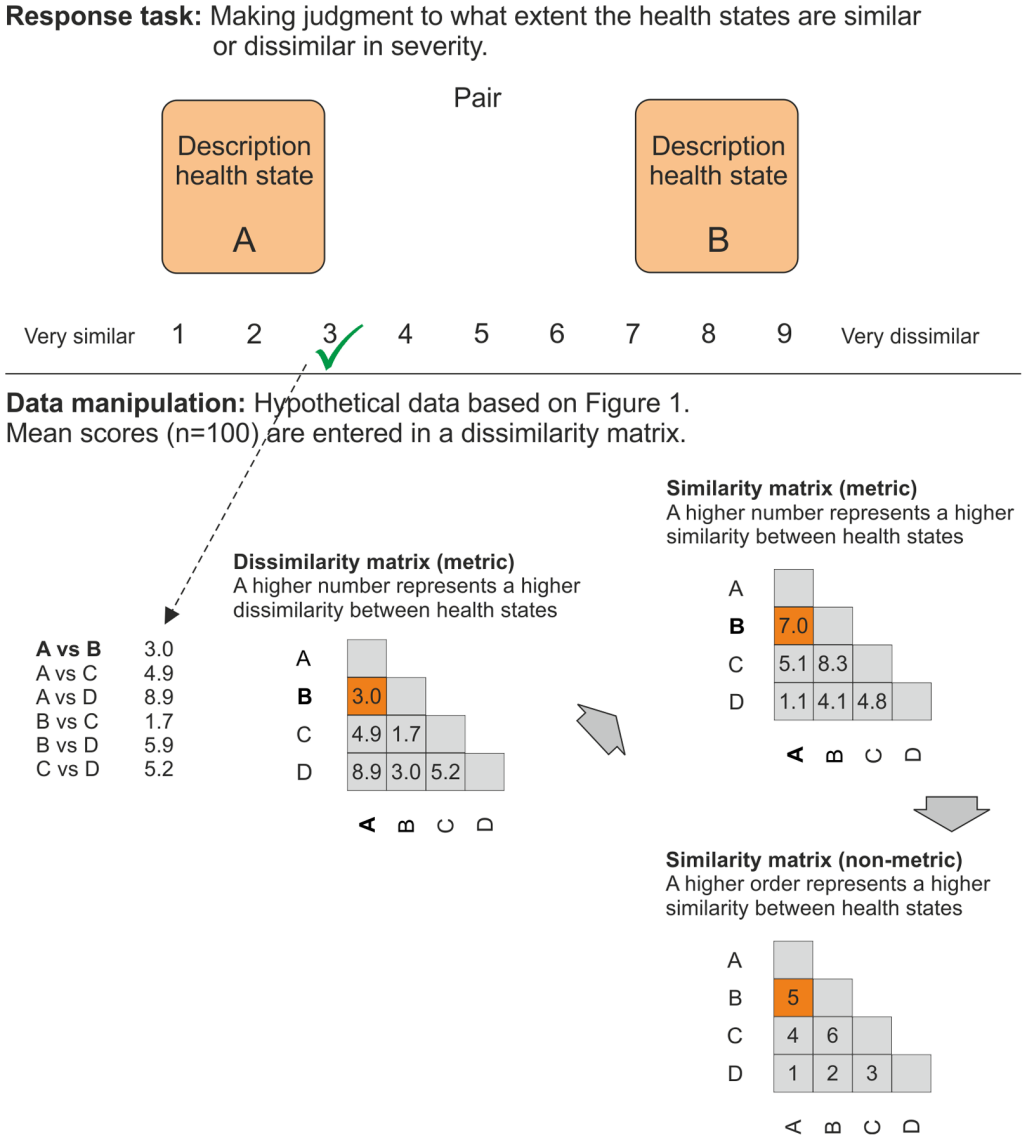
Schematic representation dyads response tasks and data manipulation.

**Figure 3:**
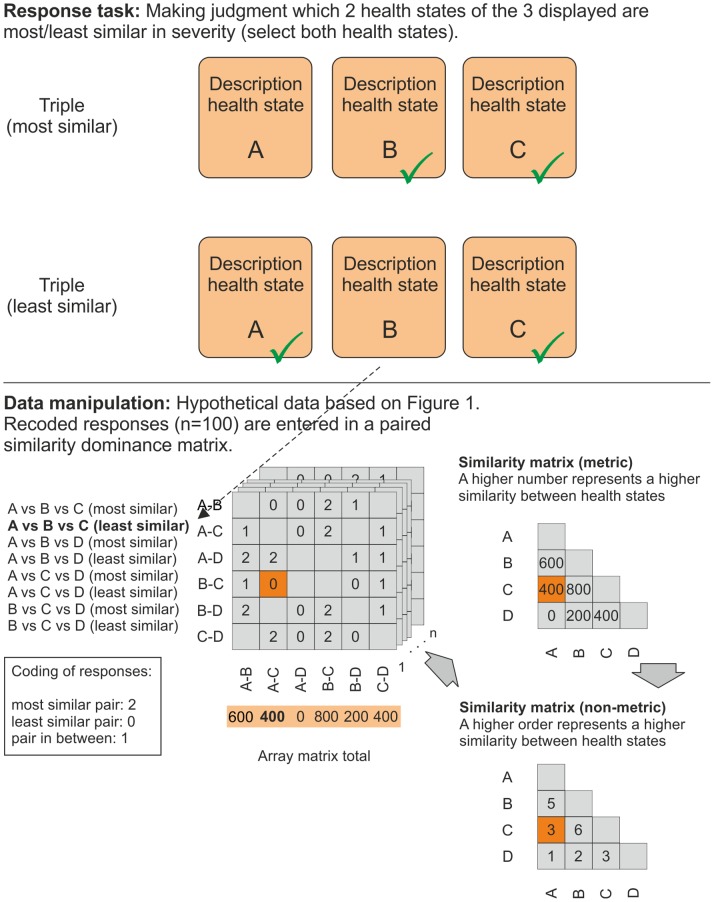
Schematic representation triads response tasks and data manipulation.

**Figure 4:**
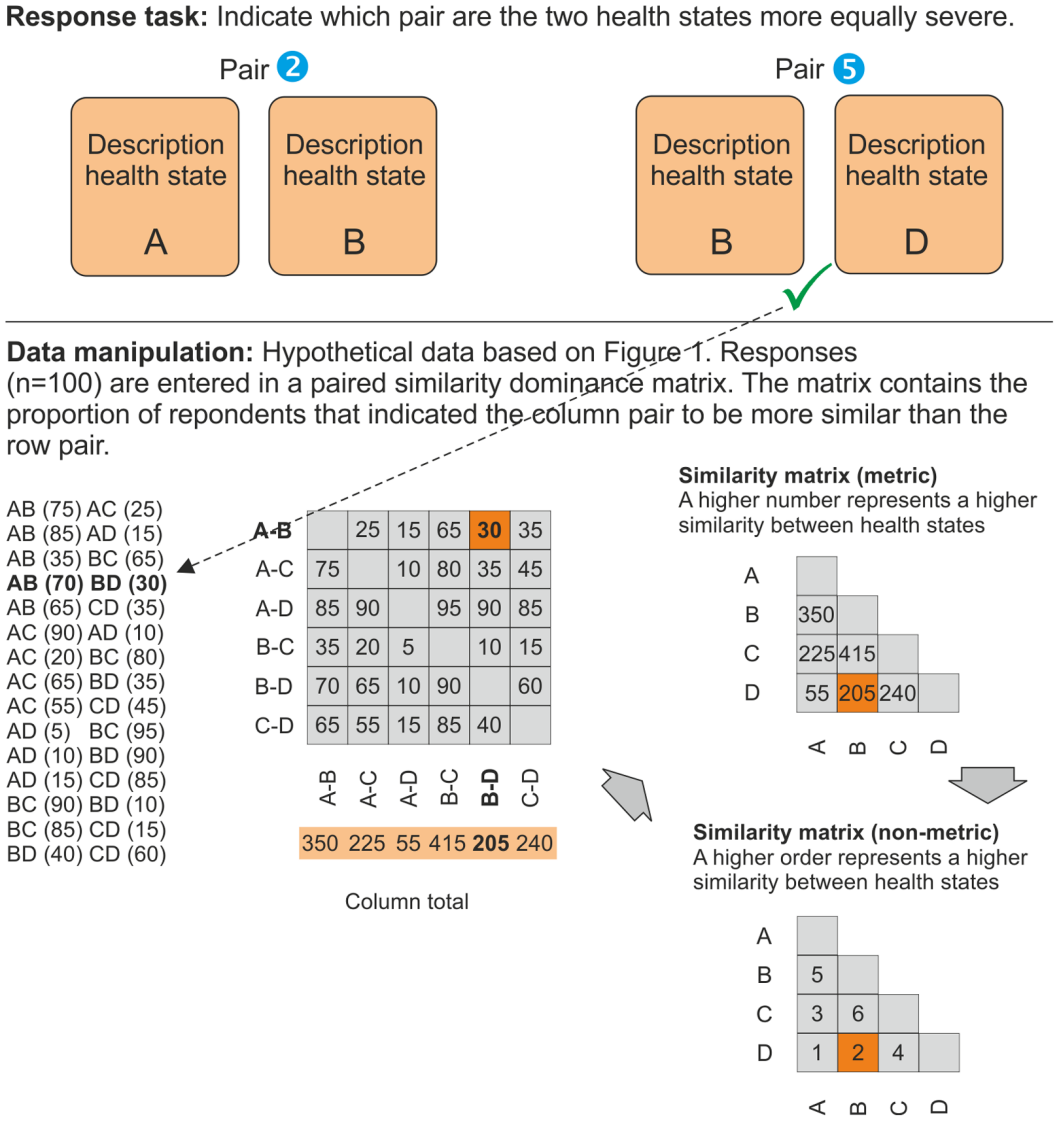
Schematic representation quads response tasks and data manipulation.

Three methods of collecting similarity data were investigated (Figures 2, 3, and 4). The first method (dyads) had a paired comparison design, whereby each health state was paired with every other one. All respondents were thus presented with 21 pairs. The task was to rate the similarity of the presented health states on a scale of 1 to 9 where 1 indicated ‘very similar’ and 9 ‘very dissimilar’ in severity. Levels 2–8 were unlabelled.

The second method (triads) had a cartwheel design, presenting each health state along with two others [32]. For each triad, the respondents had to indicate in two separate response tasks which two of the three health states shown were the *most similar* and which the *least similar* in severity. Responses were coded as a ‘2’ for the most similar pair and ‘0’ for the least similar pair. The middle pair was inferred by transitivity and coded as a ‘1’. An incomplete block design was used to minimize the burden on the respondents. Each participant was randomly assigned to a single block. In each block, one health state was held constant to facilitate comparisons, while the other two states were systematically varied per comparison. Each block thus had 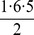 or 15 triads. Since there were two response tasks per triad, it was decided to show a respondent only half of a block. For example, block 1 had the health state ‘111111’ in each triad (16 tasks); block 2 also had the health state ‘111111’ in each triad (14 tasks); block 3 had health state ‘211111’ in each triad (16 tasks), and so on. Each respondent thus answered either 14 or 16 questions on health-state similarity. For the triads tasks, the number of inconsistencies within one triad was recorded, as inconsistencies lead to inference problems (see analyses).

The third method (quads) had a paired comparison design that presented two pairs of health states (pairs of pairs/tetrads) in each question. The respondents were asked, “In which pair are the two health states more equally severe?” Because the number of tetrads was 
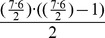
 or 210, it was decided to show each respondent 15 random choice options.

### Judgmental Processes

For the dyads response task we assumed the following judgmental processes to occur: each health state is valued independently. We define *U_ij_* as the value that respondent *i* attaches to health state *j* where U is unidimensional and composed of systematic components and unobservable components [25]

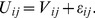
(1)


The systematic component of the value is based on a function of the combination the attributes (in this application, the DQI domains: *α*) and levels (the amount of problems on a domain: *λ*). In mathematical terms:
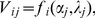
(2)where 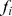 is unknown. Subsequently, the difference in value between health states *j* and *k* presented in set S is evaluated. In mathematical terms:



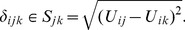
(3)Finally the difference in value between both health states is assigned an integer (*R*) on the response scale by respondents, whereby the ordinal relationship between comparisons across health-state sets is maintained. In mathematical terms:
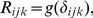
(4)where 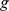 is monotonically decreasing.

For the triads response tasks we define the following judgmental processes to occur: each health state is valued independently (equation 1). Subsequently, the difference in values of health states *j*, *k*, and *l* in set *S* are evaluated:
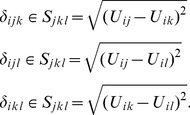
(5)


Next, respondents choose the two health states in the set that have the highest similarity.

In the second triad response task the process is reversed. Thus respondents choose the two health states that have the lowest similarity. In theory respondents re-assign values to each of the health states in this second task, which because of the error term could cause reversals in rankings. However, because this would lead to problems in estimation of the MDS scale, as we would not be able to infer a rank order, we coded responses as if the assignment of values to health states occurred once, and was stable over response tasks (see analyses).

For the quads response task we define the following judgmental processes to occur: within each pair of health states, each health state is valued independently (equation 1). Subsequently, within each pair the similarity between health states is evaluated (equation 3). Finally, respondents choose the pair with the highest similarity.

### Analyses

For the dyads method, mean dissimilarity scores were calculated with the responses on the 9-point scale and used as input for a metric dissimilarity matrix ***D***. Subsequently, this matrix was transformed into a metric similarity matrix ***D’*** by transforming each element **x**_jk_ (representing the mean dissimilarity between the column *j* and row *k* health states) in ***D*** by 10 − **x**_jk_ (see Figure 2 for the analytical process).

For the triads method, individual responses were entered in a paired similarity dominance matrix. All individual matrices were summed to construct a paired similarity dominance array. By summation over the matrices of this array, the marginals were used as input for a metric similarity matrix ***T*** (see Figure 3 for the analytical process). All inconsistent responses per triad were omitted from the analyses. Spearman correlation coefficients between the number of inconsistencies and respondents’ characteristics were calculated to assess which factors contributed to inconsistencies.

For the quads method, the percentage of times a pair of health states was chosen over another pair of health states was used as input for the paired similarity dominance matrix. In a fashion resembling the triads method, this matrix was transformed into a metric similarity matrix ***Q*** (see Figure 4 for the analytical process).

All of the above similarities were scaled with metric and non-metric MDS by means of the SPSS (version 20) algorithms in PROXSCAL [31] and rescaled to a 0–10 scale. For goodness of fit of the six solutions, the stress-1 values were compared in combination with Shepard diagrams [36]. We adopted Kruskal’s benchmark values of stress-1 values for non-metric MDS: .20 = poor; .10 = fair; .05 = good; .025 = excellent [34]. Stress values should be regarded as a badness-of-fit measure. Raw stress is defined as the sum of the squared representation errors (observed distances minus modeled distances). Stress-1 values are the raw stress values normalized for the MDS space, which is the sum of the squared distances. Stress-1 values are minimized with the following loss function:
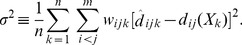
(6)


This function provides nonnegative, monotonically non-decreasing values for the transformed proximities (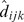). The distances (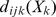) are the Euclidean distances between the health states in the rows of 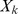 (the coordinate space). Furthermore, in the equation above 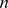 represents the number of respondents, 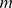 the number of health states, and 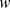 the weight given to each individual matrix (in this study always set to 1). The Shepard diagram comprises two juxtaposed plots. The first part consists of a scatter plot with proximities (observed data) on the horizontal axis and distances (model values) on the vertical axis. In metric scaling, we also have the transformed proximities that are computed by linear regression. In the Shepard diagram, the transformed proximities are added to the vertical axis and used to draw the best-fitting step function through the scatter plot of proximities and transformed proximities. Therefore, the Shepard diagram can be used to inspect both the residuals (misfit) of the MDS solution and the transformation. Outliers can be detected as well as possible systematic deviations. In non-metric MDS the transformed proximities are computed by monotone regression and are represented by a best-fitting monotone step function in the Shepard diagram.

Feasibility for each task was assessed by a 1–5 difficulty question where 1 was labelled ‘very easy’ and 5 ‘very difficult’. In addition, the median time to complete per response task and the percentage of respondents who did not finish the survey were compared.

## Results

### Respondents

In total 1510 respondents were included in the study: 532 for dyads, 469 for triads, and 509 for quads. All three samples are roughly representative for the Dutch general population in terms of gender, age, and education, although the triads sample has a skewed gender distribution (Table 1).

**Table 1:** Descriptive statistics (%) of the 3 samples that performed the 3 similarity tasks.

	Dyads(n = 532)	Triads (n = 469)	Quads (n = 509)	
Gender				
Male	51.9	34.1	49.9	
Female	48.1	65.9	50.1	
Age				
18–24	16.2	16.8	13.2	
25–34	17.3	17.5	17.5	
35–44	22.2	18.1	20.6	
45–54	22.0	24.3	25.9	
55–64	20.5	21.3	20.4	
65–74	1.9	1.9	2.4	
Education				
Low	29.9	27.3	30.1	
Medium	43.4	44.5	42.8	
High	26.7	29.2	27.1	

### MDS Solutions

#### Metric

**Figure 5:**
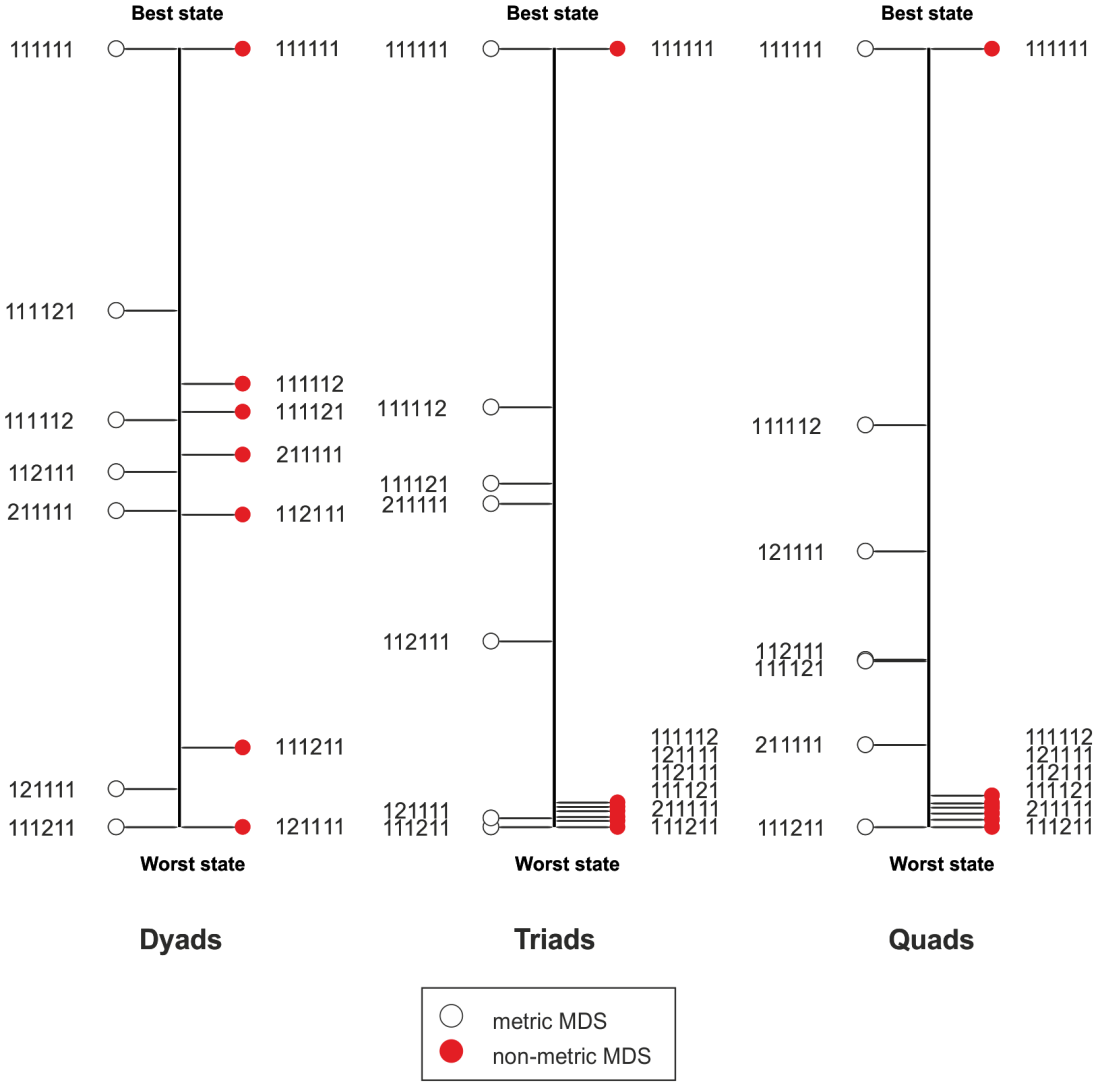
Three types of similarity judgment tasks scaled with metric and non-metric MDS.

**Figure 6:**
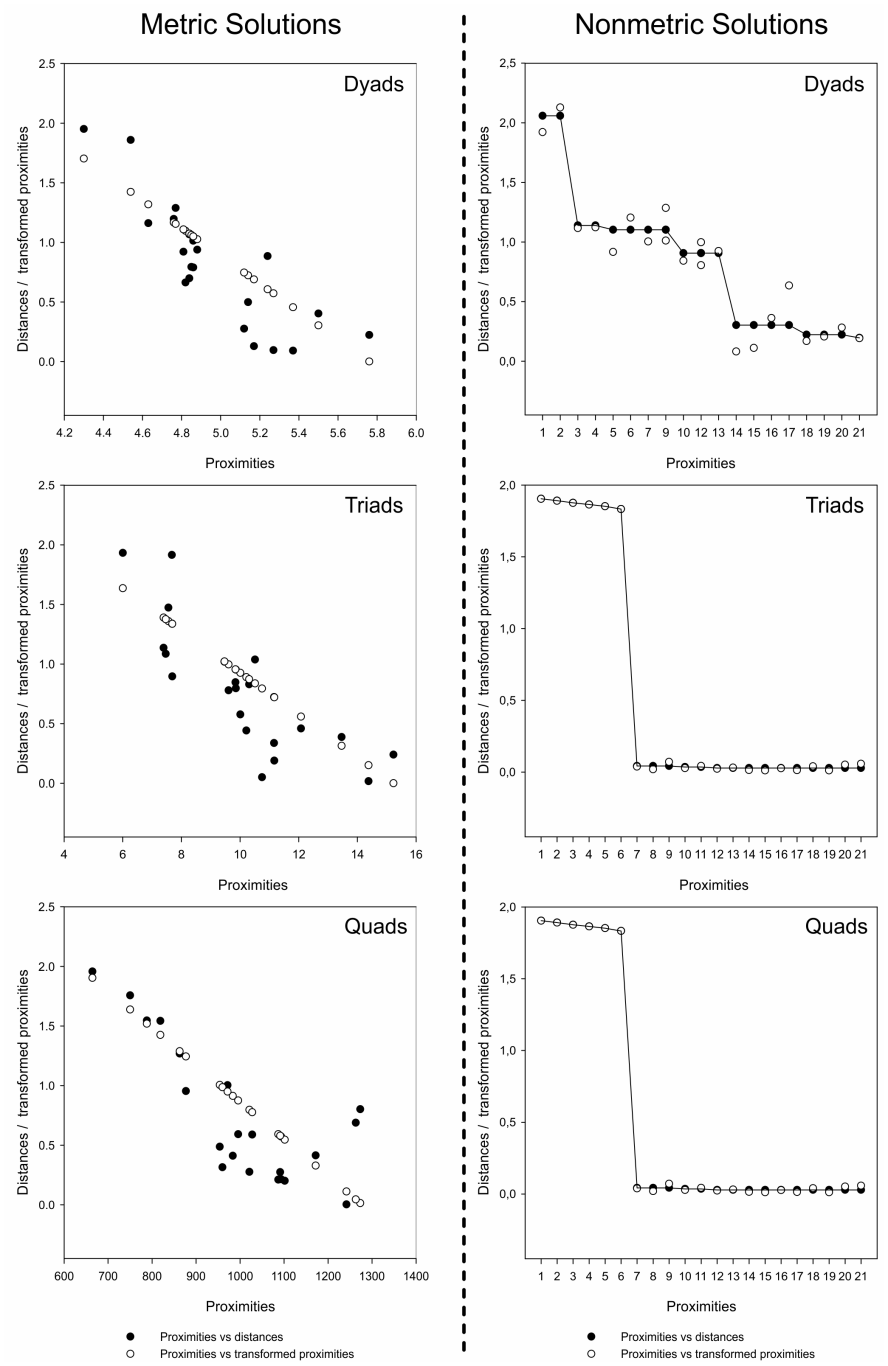
Shepard diagrams for the dyads, triads, and quads metric and non-metric solutions.

The three rescaled metric MDS solutions resulted in different rank orders for the seven health states (Figure 5). The stress-1 values for the dyads, triads, and quads solutions were 0.300, 0.331, and 0.378 respectively, indicating a poor fit [31], which is also displayed in the Shepard diagrams (Figure 6). The correlation between dyads and triads was *r* = 0.95 (*p*<0.01), between dyads and quads *r* = 0.73 (*p* = 0.063), and between triads and quads *r* = 0.81 (*p*<0.05), indicating strong to near perfect associations.

#### Non-metric

As with the metric solutions, the three rescaled non-metric MDS solutions resulted in different rank orders for the seven health states (Figure 5). The stress-1 values for the dyads, triads, and quads solutions were 0.129, 0.012, and 0.014 respectively, indicating a poor fit for the dyads but an excellent fit for the triads and quads (Figure 6). The correlation between dyads and triads was *r* = 0.76 (*p*<0.05), between dyads and quads *r* = 0.76 (*p*<0.05), and between triads and quads *r* = 1.00 (*p*<0.001), indicating strong to perfect associations.

### Inconsistencies

There were statistically significant (p<0.05) Spearman-rank correlations between the number of inconsistencies and respondents’ characteristics. These characteristics were gender (r = −0.201), education (r = −0.180), self-assessed physical health (r = 0.093), self-assessed self-care (r = 0.013), and time to complete (in seconds) (r = −0.214). This suggests that males make more inconsistent responses, as do people with a lower education, people with more problems on physical health or self-care, and people who have a lower time to complete.

### Feasibility

There were no apparent differences in the reported difficulty of the response tasks. Of the dyads, triads, and quads respondents, 26%, 29%, and 29% respectively found the task (very) easy, while 31%, 31%, and 30% found it (very) difficult. The median times to complete per choice task were 11.7, 20.5, and 17.1 seconds for the dyads, triads, and quads respectively. The number of drop-outs was 8%, 19%, and 7% for the dyads, triads, and quads respondents respectively. In the triads task, the percentage of respondents who had one inconsistency in at least one triad was 48%.

## Discussion

This is the first explorative study attempting to demonstrate the feasibility of eliciting similarity data on health states and scaling these data with metric and non-metric multidimensional scaling (MDS). One of the main motives to investigate MDS was the fact that choice models suffers from dominance problems at the top and the bottom of the health-state continuum. Similarity judgments do not have this limitation. In fact, combining similarity response tasks with conventional choice tasks may be an attractive strategy.

Three different response tasks to elicit similarity data were investigated. All three provided data that was scaled with MDS in such a way that health state ‘111111’ was positioned at the end of the HRQoL continuum. This is a logical requirement of any health-state continuum and serves as a validity check of the derived data. Additionally, all MDS solutions based on the three response tasks had state ‘111211’ positioned at the end of the scale.

Interestingly, there were discrepancies between the three tasks for the health states in between. These are difficult to explain from a theoretical point of view. In regard to the fit statistics, the triad and quad similarity responses were scaled excellently with non-metric MDS. These solutions had a similar rank order and perfect association. However, all non-optimal states were clustered together. This would indicate that respondents do not perceive a substantial difference in quality between health states with some problems on one domain, which is consistent with previous findings based on preference data [39]. Given the content of the Shepard diagrams, a more likely explanation is that the non-metric solutions were degenerative. A degenerative solution occurs when fit statistics approach zero, even though the data are not represented properly. What we observed is a dichotomization of the data. One cluster of distances between ‘perfect health’ and the other six health states, and a cluster of distances between all pairs of health states in which all states have some problems on a single domain. In such a solution, only one aspect is properly represented: the ‘between-block’ distances are larger than the ‘within-block’ distances. As Borg & Groenen [31] state: “This type of degeneracy can be expected with ordinal MDS when the dimensionality is high compared to the number of objects. It all depends though, on how many within-blocks of zero exist.” In the current study the number of dimensions was one, the number of objects seven, and the number of within-blocks of zero was two. It is this last aspect that we clearly observe in the Shepard diagrams. To avoid degeneracy, stronger restrictions can be imposed. Examples of such restrictions are linear transformations with an intercept, spline transformations, or any other type of metric representation. Since we investigated metric as well as non-metric MDS, we have already imposed metric restrictions. These did not represent the data very well, as indicated by the Shepard diagrams and fit statistics.

A benefit of the MDS models is that interval-level data as well as ordinal-level data can be used to generate metric scales. An example of a comparison between metric and non-metric MDS in an application of health-state valuation can be found in the study by Krabbe et al. [30] In this study distances between health states were derived by summing the squared distances of empirically obtained VAS values and then taking the square root of the sum. Assuming the VAS obtains interval-level data, these distances also have interval-level properties, and can thus be scaled with metric MDS. Almost exactly the same distances were scaled with non-metric MDS by assigning integer ranks to each of the distances. The Spearman rank correlation coefficient between the metric and non-metric MDS solutions was close to 1.0. These results were not surprising as the number of ordinal constraints (i.e. 171 similarities) was sufficiently high. An earlier study by Shepard [38] investigated this same issue in a different context. Shepard used Monte Carlo simulations to reconstruct random points in a two-dimensional space with non-metric MDS. He found that for 7 points the root-mean-square of the 7 correlations was 0.969 between the true distances and the nonmetric MDS solution. When the number of points increased to 45 this correlation was as high as 0.99999994.

The triads response tasks resulted in at least one inconsistency for nearly half of the respondents, which casts doubt on the feasibility of this response task. It suggests that internet surveys are not an appropriate medium for this response task. However, such an interpretation seems groundless, since respondent answers on the feasibility question do not suggest such a high level of inconsistent responses. The dyads and quads task had the lowest number of people dropping out. These two tasks appear to be the most promising for eliciting health-state similarity judgments in an online setting.

This study produced different results for each of the three similarity response tasks. Possible explanations for these differences are the following. The solutions are based on a relatively low number of similarities. Since only seven health states were used, the number of similarities was 21, which might be too low. Probably more relevant is the fact that the health states that were chosen in the current study turned out to be quite similar in severity. When the methodology for this study was discussed, no health-state values for the 6 DQI states with ‘some problems’ on a domain were available. In a later DC experiment [39] performed on DQI health states, the regression coefficients for each of the domain-levels ‘some problems with…’ showed overlapping confidence intervals. Therefore, it seems likely that the current study focuses on a very narrow space on the health-state continuum. We recommend that future studies use a more diverse set of health states that would cover a broader range than used in this study or even cover the entire health-state continuum. Another consideration is to use health states from well-established value-based classification systems such as the EQ-5D [40]. This would allow for more inferences of validity of MDS by comparing it with TTO and DC models.

One strength of the current study is the large number of respondents. The total sample was representative of the Dutch general population in terms of gender, age, and education. What we did not take into account is that MDS is able to cope with missing data. If the error level is low, excellent representation is possible with as much as 80% missing data, provided the data is scaled in the ‘true’ dimensionality and that the number of health states is high compared to the number of dimensions [31]. This allows for less-demanding incomplete designs to be used in future studies.

Eliciting similarity data also has some limitations. The number of respondents required to obtain similarity data is higher than for preference-based data (e.g. TTO and DC). At present, there is no indication which combination of health-state pairs will provide the most optimal similarity matrices. Another limitation is that the process of aggregating individual data into similarity matrices is non-standardized. Furthermore, the MDS approach uses response tasks that are potentially more difficult than a single DC task.

Despite the abovementioned limitations the MDS approach could be advantageous compared to other valuation methods. From a theoretical point of view the MDS approach compares favorably to both TTO and SG as the judgmental task is not influenced by problems such as adaptation, discounting, time preferences, a choice for indifference procedures, nor are there difficulties quantifying states considered worse than ‘dead’.

Compared to DC models, which also do not suffer from the abovementioned limitations, the MDS approach offers some additional advantages. Currently there are limitations regarding scaling DC models on the dead–perfect health scale [41], although attempts to overcome this limitation have been put forward [42]. Additionally, in DC models researchers have to make assumptions and choices regarding the functional form of the value function (e.g., only main effects or main effect and interactions, or a multiplicative model instead of a linear model). In the MDS models, the functional form of the value function is undefined, allowing for more realism and full flexibility in respondent heterogeneity.

There are several options to use similarity data to arrive at a full set of values for all possible health states. For the dyads task individual similarity matrices can be obtained. These matrices can be scaled to obtain health-state values for each state present. Similar to valuation studies using TTO or SG, by using regression techniques health-state values can be estimated for the health states not included in the dyads tasks. For the triads and quads methods the possibilities are more restricted. If we want to derive values for all health states of a particular value-based system (e.g., DQI, EQ-5D), then a matrix that contains similarity judgments on all health states is required. This seems an extremely challenging task as it could require millions of similarity responses. Future work should address this particular issue and investigate avenues to overcome this limitation. One possible solution that has been put forward for another novel health-state valuation method [43] is to include similarity response tasks as a standardized part of (inter)national health surveys. In time this could lead to a sufficient amount of data to estimate values for all possible health states of a particular health-state classification system. Another way of applying MDS is by transforming preference data (e.g. TTO, DC) into similarity data. Nevertheless, this methodology would still suffer from dominant choice sets.

Since similarity data have the biggest potential for scaling data to a one-dimensional interval-level scale based on a single response task, the above-mentioned suggestions and limitations point to fruitful directions for future research in the field of health-state valuation.
